# Beneficial effects of nicotinamide on the mouse model of preeclampsia

**DOI:** 10.33118/oaj.preg.2019.01.002

**Published:** 2018-11-26

**Authors:** Phillip K Huynh, Nobuyuki Takahashi, Nobuyo Maeda-Smithies, Feng Li

**Affiliations:** 1 Dept of Pathology and Laboratory Medicine, The University of North Carolina, USA.; 2 Division of Clinical Pharmacology and Therapeutics, Tohoku University Graduate School of Pharmaceutical Sciences and Faculty of Pharmaceutical Sciences, Sendai, Japan.; 3 Dept of Medicine, Div. of Nephrology, Endocrinology, and Vascular Medicine, Tohoku University, Sendai, Japan.; 4 Dept of Cell and Molecular Physiology, The University of North Carolina, USA.

**Keywords:** Preeclampsia, Nicotinamide

## Abstract

Preeclampsia (PE) is a pregnancy related disorder that is characterized by hypertension and proteinuria in the mother. It is associated with impaired coagulation and liver function, and a variety of other detrimental effects. In severe cases without treatment, PE can progress to eclampsia and result in seizures, a life-threatening condition. Although the etiology of PE is largely unknown, sFlt-1 (soluble vascular endothelial growth factor receptor 1) released by the impaired placenta resulting from insufficient perfusion plays a critical role in PE, and phenotypes of PE can be induced by experimentally increasing sFlt-1. We and other investigators have proposed that endothelin-1 (ET-1) system is the mediator of the pathological effects of excess sFlt-1, and antagonists of ET-1 receptor block the effects of sFlt-1. Unfortunately, this class of drugs is teratogenic and unsuitable for treating pregnant women.

Nicotinamide is a naturally occurring derivative of vitamin B3 in the body and inhibits ADP-ribosyl cyclase, which is activated by the ET-1 receptor. Therefore, if utilized, it would be expected to play a beneficial role in PE. In mouse models of PE, a high dose of nicotinamide shows great success in lowering blood pressure, correcting renal function and structure, prolonging pregnancy as well as increasing fetal weight/number. Nicotinamide, being generally regarded as safe, could be a promising substance to further investigate for use in clinical trials.

## Preeclampsia

Preeclampsia (PE) is a hypertensive disorder unique to pregnancy that occurs in approximately 5–8% of U.S. pregnancies [1,2]. In the United States, the incidence has dramatically increased in the past three decades [3,4]. The disease is mild in 75% of cases, but is severe in the 10% of cases that occur before 34 weeks of gestation [5]. PE is a leading cause of maternal morbidity and mortality, and women who develop PE are at risk for pulmonary edema [6], coagulation defects, hepatic and/or renal failure, seizures, cerebral hemorrhage, visual disturbance [7-10], and death [11]. PE is also a significant contributor to neonatal morbidity and mortality. Fetal growth restriction, probably due to chronic placental hypoperfusion, is a common complication of PE [12]. Women with PE are 3–4 times more likely to deliver small-for-gestational age babies compared to women with normal pregnancy [13]. Placental abruption, premature separation of the placenta with disruption of blood flow to the fetus, is an uncommon but dangerous complication that occurs in up to 3% of pregnancies complicated by severe PE [14]. Some biomarker measurements during the first trimester, such as serum pregnancy-associated plasma protein-A and placental growth factor, combined with maternal history, characteristics and non-invasive methods to measure placental vascularization indices, can identify a high proportion of early onset PE [15-17], but further research is required to predict which women could develop PE in general and how severe it could become. The existing treatment options for PE are limited [18], although a low dose aspirin administered before 16 wk of pregnancy had beneficial effects on prevention of morbidity and mortality of PE based on a recent meta-analysis of a large numbers of clinical trials [2]. PE-related care had cost the U.S. health care system more than $2 billion in 2012 alone [18]. Clearly, it is urgent to develop prophylactic/therapeutic strategies to safely postpone delivery for the infant’s benefit without increasing maternal risk. Here we discuss the potential benefits of nicotinamide in PE.

## Endothelin-1 and Preeclampsia

While the initiating causes of PE remain elusive, it most likely starts when placentation is impaired, leading to poor perfusion and the release of soluble anti-angiogenic factors [5]. What determines the severity of PE symptoms in mothers is still not clear, although recent clinical and experimental observations suggest that the soluble anti-angiogenic factor, sFlt-1 (sVEGFR-1), is responsible for many of the maternal manifestations of PE that include hypertension, proteinuria, and end organ damage. Indeed, plasma levels of sFlt-1 correlate with the severity of the hypertension [19]. These maternal clinical signs can be replicated in rodents by increasing circulating sFlt-1 experimentally [20-22]. Furthermore, we and other investigators have reported that the endothelin-1 (ET-1) system in mothers is activated when sFLt-1 levels in vivo are increased experimentally, and PE-like phenotypes are ameliorated by antagonists of ET-1 receptor. These observations have led to a notion that ET-1 is a mediator of pathophysiological effects of sFlt-1 [21-23].

ET-1 is a 21 amino acid proteolytic product of preproET-1 coded by the *EDN1* gene. Although there are evidences that ET-1 is a potent vasoconstrictor and involved in hypertension and kidney damage [24,25], the exact mechanism of ET-1 regulating blood pressure (BP) is very complex. For example, the mice carrying one null-mutant allele and one wild type (WT) allele of *Edn1,* which produce lower levels of ET-1 than WT mice, have elevated BP [26]. On the other hand, ET-1 transgenic mice with higher than normal ET-1 level have normal BP [25]. The complex of ET-1 system is elegantly reviewed by Speed *et al.* [27].

Aggarwal *et al.* reported an association between *EDN1* G5665T polymorphism with elevated plasma ET-1 and with PE. They observed that women with PE had an increased frequency of the T-5665 allele and circulating ET-1 levels, and maternal *EDN1* genotype was correlated with the severity of hypertension in PE [28]. Accordingly, we examined the effects of pregnancy in *Edn1*^*H*/+^ mice that carry a modified *Edn1*^*H*^allele that increases its mRNA stability and thus have four times normaL levels of ET-1 expression [29]. *Edn1*^*H*/+^ females (mated with wild type males) develop PE- like phenotypes including a marked increase in BP, proteinuria and kidney damage during the third week of pregnancy despite showing no observable abnormalities before the pregnancy [30]. Our data demonstrated that maternal overexpression of ET-1 is sufficient to cause the end organ damage of PE, confirming that it is the operational status of the entire ET-1 system that predisposes mothers to PE.

## Nicotinamide as a promising new agent to benefit Preeclampsia

**Figure 1.**
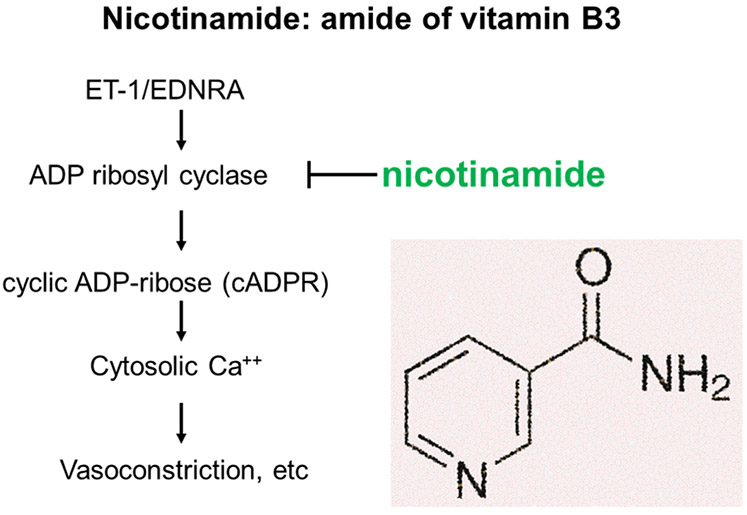
Nicotinamide inhibits ADP ribosyl cyclase which is activated by ET-1.

ET-1 exerts its biological functions via binding to ET receptors: type A and B receptors (EDNRA and EDNRB). Usually, EDNRA is involved in vasoconstriction [27]. Accordingly, EDNRA antagonists, such as ambrisentan, blunt its actions and greatly decrease the severity of the PE-Like phenotype induced by increased sFlt-1 [21, 22]. Unfortunately, this class of drugs is teratogenic and consequentlfetotoxicy unacceptable for use in treating PE [31]. However, this does not preclude the use of substances that block the action of factors downstream of the EDNRA, provided that these substances are not fetotoxic. Nicotinamide (amide form of vitamin B3: Nam) inhibits ADP-ribosyl cyclase, which is activated via the EDNRA (Figure 1) [32]. Nicotinamide is promising because, unlike EDNRA antagonists, it is generally regarded safe for use in pregnancy [33].

Li *et al.* therefore tested this agent in two different mouse models of PE: one with sFlt-1 overexpression induced by adenoviral vector (AdV) administration, and another with genetically lacking ASB4 (Ankiryn-repeat-and-SOCS-box-containing-protein). The study found that nicotinamide given in their drinking water at a dose of 500mg/kg/day at 12.5 dpc (day post coitus, 2 days before the virus administration) has beneficial effects on both mothers and fetuses [34]. In the first model, nicotinamide decreased the elevated systolic BP (SBP) and urinary albumin excretion induced by excess sFlt-1. Histological examination showed that sFlt-1 causes severe endotheliosis in kidneys, and nicotinamide significantly improves kidney structures in these mice. Nicotinamide also prolonged the duration of pregnancy and increased fetal body weights. In the second model, nicotinamide treatment started at the first day of pregnancy also executed beneficial effects on dams with impaired placental development caused by genetic lack of ASB4 [34]. Nicotinamide corrected the hypertension and albuminuria which present in 3rd week of pregnancy in *Asb4*^−/−^ dams, and it also ameliorated their pregnancy-associated changes of the kidney structures. Nicotinamide also increased the duration of pregnancy by approximately one day, and prevented embryo/fetal loss in *Asb4*^−/−^ dams. In the second report, Fushima *et al.* tested the effects of nicotinamide on the third mouse model in which the PE-like phenotype was induced by a surgical reduction of uterine perfusion pressure (RUPP). Nicotinamide also improved maternal hypertension, proteinuria, and glomerular endotheliosis, prolonged pregnancies and improved survival and growth of the embryos in RUPP mice [35].

Nicotinamide increases the content of embryo/fetal ATP and nicotinamide adenine dinucleotide (NAD) in all the three PE models, suggesting that the beneficial effects of nicotinamide on embryos/ fetuses are probably through normalizing embryonic/fetal ATP synthesis via the nucleotide salvage pathway. The study of niacin (vitamin B3) supplement conducted by Shi *et al.* provides an additional evidence that dietary vitamin B3 improves the pregnancy outcomes. They identified variants in two genes, *HAAO* and *KYNU,* that encode 3-hydroxyanthranilic acid 3,4-dioxygenase and kynureninase, respectively, in families in which a person had multiple congenital malformations. These enzymes in the kynurenine pathway are essential for the de novo synthesis of NAD from tryptophan. They examined pregnancy in mice with null mutations in these two genes and showed that all the fetuses from *Haao*^−/−^ or *Kynu*^−/−^ dams were lost when niacin was depleted in the maternal diet during the pregnancy. In contrast, niacin supplemented in their drinking water at 15mg/L completely prevented the malformation and resorption by normalizing the energy production in fetuses [36]. NAD plays a fundamental role in cell metabolism by carrying electron for redox reactions during energy metabolism. It is also essential in pathways for biosynthesis of purine and pyrimidine nucleotides. The finding that a relatively small amount of niacin supplementation in diet completely ameliorated the congenital malformations in the deficiencies of de novo NAD biosynthesis is of interest. However, whether or not the dams with these mutations develop PE like symptoms were not reported.

## Summary and Perspectives

The studies in rodent models thus clearly demonstrate that nicotinamide has the potential to reverse serious maternal sequelae of PE to allow prolongation of pregnancy and improve infant outcomes, regardless of the cause that leads to PE. While translation of the findings made in mice to humans is not a simple issue, it is important to test the effects of this vitamin in humans because there is no real treatment available, and because even a few more days of intra-uterine life for the babies of human patients with early-onset PE would have real benefits.

**Figure 2.**
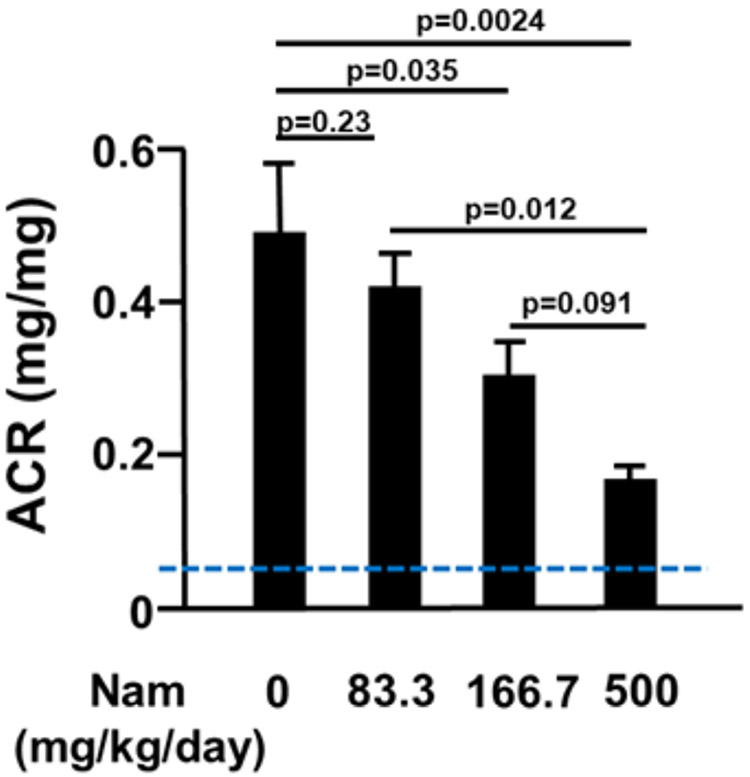
The effects of different doses of nicotinamide (Nam) on urinary albumin excretion in C57BL/6J WT virgin female mice received 1×10^9^PFU of sFlt-1-AdV. After injection with sFlt-1-AdV, mice were randomly enrolled into four groups and treated with varied amounts of Nam via oral gavage daily. Average albumin/creatinine ratios (ACR) in urine collected at 7 days after the virus injection is shown as mean+/−SEM. The blue dashed line indicates the normal level of ACR in mice without sFlt-1-AdV. N=5 per group.

Meanwhile there are many aspects of nicotinamide treatments of PE that need to be further investigated in mouse models. For example, the selected dose (500 mg/kg body weight/day) used for mice in our experiment was based on the effects to decrease the elevation of albumin/creatinine ratio when nonpregnant WT mice were treated with adenovirus to overproduce sFlt-1 as shown in Figure 2 (unpublished data). There have been no adverse effects observed at this dose at any stages of pregnancy in mice. The dose is equivalent to 2.5 g/day for a 60 kg woman when corrected for body surface area. This is less than 3 g/day that have been employed in other trials of diabetes interventions [33,37]. However, although the risk of nicotinamide toxicity appears to be quite low, the upper limit during pregnancy in humans has not been reported. On the other hand, nicotinamide at dose 167mg/kg/day significantly decreased the elevated urinary albumin excretion induced by sFlt-1 in the mice in the experiment in Figure 2, suggesting that one third of the dose of nicotinamide we have been using (equivalent to less than 1 g/day for a 60kg woman) could also have beneficial effects in PE.

The second aspect is that we lack knowledge regarding when exactly the nicotinamide shows the key therapeutic effects for PE. A key question to ask is whether or not the maternal PE symptoms would be ameliorated when the dams are treated with nicotinamide after PE is diagnosed during the third week of pregnancy, or would supplementation have to be given earlier during early implantation stage or when placental growth is at its peak. This would simultaneously clarify the pathogenesis of PE. Clinically, the increase in circulating sFlt-1 levels precedes the manifestation of PE symptoms. Therefore, it is worthwhile to test the effects of nicotinamide treatment starting at different gestational stages and for different durations of the pregnancy.
